# COVID‐19 outcomes among patients with dementia and age‐matched controls who were hospitalized in 21 US health‐care systems

**DOI:** 10.1002/alz.14136

**Published:** 2024-07-29

**Authors:** Adrienne L. Johnson, Nathaniel A. Chin, Thomas M. Piasecki, Karen L. Conner, Timothy B. Baker, Michael C. Fiore, Wendy S. Slutske

**Affiliations:** ^1^ Center for Tobacco Research and Intervention School of Medicine and Public Health University of Wisconsin Madison Wisconsin USA; ^2^ Department of Medicine School of Medicine and Public Health University of Wisconsin Madison Wisconsin USA; ^3^ Wisconsin Alzheimer's Disease Research Center University of Wisconsin School of Medicine and Public Health Madison Wisconsin USA; ^4^ Division of Geriatrics Department of Medicine School of Medicine and Public Health University of Wisconsin Madison Wisconsin USA; ^5^ Department of Family Medicine and Community Health School of Medicine and Public Health University of Wisconsin Madison Wisconsin USA

**Keywords:** Alzheimer's disease, COVID‐19, dementia, electronic health records, mortality, vascular dementia

## Abstract

**INTRODUCTION:**

COVID‐19 had devastating impacts worldwide. However, most research examining the impact of dementia on COVID‐19 outcomes has been conducted in Europe and Asia and has not examined dementia subtypes.

**METHODS:**

A retrospective analysis of electronic health record data from 21 US health‐care systems examined relationships of all‐cause dementia, Alzheimer's disease (AD), and vascular dementia with in‐hospital mortality, intensive care unit (ICU) admission, and hospital stay duration.

**RESULTS:**

All‐cause dementia, but not AD or vascular dementia independently, was associated with increased mortality risk, the inclusion of discharge to hospice as a mortality equivalent increased risk for mortality for all‐cause dementia, and AD and vascular dementia. Patients with all‐cause dementia and AD were less likely to be admitted to the ICU than patients without. Patients with any form of dementia had longer hospital stays than patients without.

**DISCUSSION:**

Dementia was associated with increased mortality or hospice discharge, decreased ICU admissions, and longer hospital stays.

**Highlights:**

- Only all‐cause dementia was associated with increased mortality risk.
- This risk was lower than what has been published in previous research.
- Combining mortality and hospice discharge increased risk for all dementia subtypes.
- All‐cause and Alzheimer's disease (AD) dementia were associated with decreased intensive care unit admissions.
- All‐cause, vascular, and AD dementia were associated with longer hospital stays.

## 1 BACKGROUND

COVID‐19 has resulted in 768 million confirmed cases and 6.9 million deaths worldwide.^1^ In the United States, there have been 103 million confirmed COVID‐19 cases and 1.1 million deaths.^2^ Moreover, COVID‐19–related hospitalizations overwhelmed hospital capacity resulting in excess deaths.^3^ Certain groups, such as older adults and those with medical comorbidities, are at especially “high risk” of negative impacts of COVID‐19 including hospitalization, intensive care measures, and death.^4^

One COVID‐19 medical comorbidity of particular importance is dementia, due to its increased risk among older adults,^5^ shared genetic underpinning,^6^ and association with higher mortality rates.^7^, ^8^ A recent article highlighted that apolipoprotein E ε4, the strongest monogenetic risk factor for Alzheimer's disease (AD),^9^ was associated with rapid disease progression and death in COVID‐19–infected mice and higher mortality rates in humans.^6^ Two meta‐analyses found that patients with all‐cause dementia were 1.80^7^ and 5.17^8^ times more likely to die while in the hospital than patients without dementia, although mortality estimates among studies varied greatly.^8^ A meta‐regression analysis showed that the association between dementia and mortality was influenced by covariates of increasing age and comorbid cardiovascular issues (i.e., hypertension); however, these were not identified as modifiers in the dementia–mortality association.^7^ Rather, authors proposed that older age and comorbid conditions that commonly occur in later age are independent mortality risk factors; therefore, as older adults age they are more likely to die regardless of dementia presence.^7^, ^10^ Multimorbidity is a common problem in COVID^11^ cases as well as dementia cases,^12^ and may be related to the increased risk of death. A recent study supported this theory by statistically matching participants on age, sex, and medical comorbidity, there was a significant, but lowered, impact of dementia (odds ratio [OR] = 1.33) on mortality than when controlling for these variables statistically (OR = 1.84).^13^ Further, inflammation, a symptom associated with both COVID and dementia,^14^ may increase the risk of death in dementia.^15^

Although there has been significant work examining the impact of dementia on COVID‐19–related mortality, many limitations are notable. First, the vast majority of research examining the impact of COVID‐19 among dementia patients has been completed primarily in Asian and European countries.^13^, ^16^, ^17^, ^18^, ^19^, ^20^, ^21^, ^22^, ^23^, ^24^, ^25^, ^26^, ^27^, ^28^ This is problematic given the United States has the highest COVID‐19–related death count of all wealthy countries^29^, ^30^ and shows differences in hospital capacity and use.^31^ Of the studies conducted in the United States, results varied. Most studies showed an increased risk of mortality for patients with, versus without, dementia^10^, ^32^, ^33^, ^34^, ^35^, ^36^ (OR/risk ratio range = 1.2–3.6), but one study showed no significant difference between patients with, versus without, dementia.^37^ Only two studies examined intensive care unit (ICU) admission among COVID‐19–positive patients; one found no difference in ICU admission status^10^ while the other found a lower likelihood of ICU admission among patients with, versus without, dementia.^37^ ICU admission may be elevated in those with dementia due to increased sensitivity to changes among dementia patients,^38^ resulting in a lower threshold for ICU admission. The co‐occurrence of delirium^39^ in dementia patients and difficulty accurately differentiating these conditions^40^ could result in longer hospital stays. Also, dementia patients commonly have poor oral intake, which is associated with increased hospitalizations, mortality, and ICU admission.^41^, ^42^, ^43^, ^44^

Only five studies have examined the impact of different forms of dementia on COVID‐19–related outcomes,^19^, ^26^, ^27^, ^34^, ^36^ potentially explaining the noticeable heterogeneity in findings. Different dementia subtypes likely have different mechanisms of action explaining the dementia–COVID relation. However, due to limited research in this area, what these mechanisms are is less clear, and it first must be understood which dementia groups differ by hospital outcomes. Existing studies found a consistently elevated risk for mortality and hospitalization among patients with AD and all‐cause dementia,^19^, ^26^, ^27^, ^34^, ^36^ but not vascular dementia.^34^ Last, all but two studies^13^, ^34^ occurred prior to the development of vaccinations and the emergence of COVID‐19 variants, which we know altered ICU admission and mortality rates.^29^, ^30^, ^45^ There is a pressing need for research examining the impact of all‐cause dementia and the two most common forms of dementia (AD and vascular dementia)^5^ among a large and contemporary US sample.

The aim of the present study was to examine the impact of all‐cause dementia, AD, and vascular dementia among hospitalized COVID‐19–positive adult patients on COVID‐19–related severity outcomes of mortality, ICU admission status, and duration of hospital stay. Patients with and without dementia were matched based on age. We hypothesized that patients with all‐cause and AD dementia would have higher rates of mortality than patients without dementia, whereas patients with vascular dementia^34^ would show no difference in mortality risk. We further hypothesized that all‐cause dementia would show no difference in ICU admission status,^10^ but did not have specific predictions for AD or vascular dementia given the dearth of research on these subtypes. Last, we hypothesized that patients with all‐cause, AD, and vascular dementia would have longer hospital stays than patients without these diagnoses.^46^

## 2 METHODS

### 2.1 Study design

The COVID EHR Cohort at the University of Wisconsin (CEC‐UW; ClinicalTrials.gov: NCT04506528)^47^ is a retrospective cohort study funded by the National Cancer Institute that included 21 health‐care systems from across the United States (see Figure S1 in supporting information for names and locations of systems and Figure S2 in supporting information for distribution of study data by dementia type). Data extractions were performed using a customized extraction code altered to accommodate unique features of each health‐care system specific to the electronic health record (EHR). Health‐care systems provided EHR data for all COVID‐19–positive patients (defined as having a positive polymerase chain reaction [PCR] test and/or COVID International Classification of Diseases 10th Revision [ICD‐10] diagnosis [U07.1 or J12.82]) for the data collection period (February 1, 2020–January 31, 2022). Health‐care systems extracted and transferred EHR data regularly to the CEC‐UW Coordinating Center in Madison, Wisconsin, where data were harmonized and merged. Local and site‐specific institutional review board approval (or exemption status) was obtained prior to sharing any EHR data. The study was performed in accordance with the ethical standards as laid down in the 1964 Declaration of Helsinki and its later amendments or comparable ethical standards. This study ensured strong inclusion of underrepresented groups by collecting data from 21 health‐care systems across the United States, therefore addressing diversity, equity, and inclusion (DEI).

### 2.2 Cohort definition

The starting sample of 145,944 adults who were hospitalized with COVID‐19 was used to identify all patients with dementia diagnoses and age‐matched controls. To be eligible for the study, participants had to (1) be age 18 or older, (2) be hospitalized for COVID‐19 for at least 24 hours (or have died or been transferred to ICU within 24 hours of admission), (3) have a positive COVID‐19 PCR test or an ICD‐10 COVID‐19, and (4) have had prior contact with the health‐care system they were admitted to before the data collection period. Compared to the full sample,^48^ the subsample for the current study appeared older, had a higher representation of White adults, and had more females. Given the focus on dementia in the current article, these subsample differences were not examined further as participants were matched on a 1:1 basis to controls (see Primary analyses section).

This study focused on three separate samples and respective matched counterparts. Groups included all‐cause dementia (ICD‐10 F01, F02, F03, G30, G31.0, G31.01, G31.09, G31.1, or G31.83; *N* = 11647), AD (ICD‐10 G30; *N* = 2648), and vascular dementia (ICD‐10 F01; *N* = 2438). ICD‐10 codes used to determine groups were required to be present at either admission, discharge, or encounter‐level data. See supporting information for frequencies of dementia subtypes for all‐cause dementia. Groups were matched on a 1:1 basis based on age (± 5 years) and matched controls did not hold an ICD‐10 diagnosis for any type of dementia.

### 2.3 Primary outcomes

The primary outcomes included mortality in hospital during COVID‐19–related hospitalization, admission to the ICU while in the hospital, and hospital stay duration (among patients who did not die prior to discharge).

**RESEARCH IN CONTEXT**

1. **Systematic review**: The authors reviewed the literature using traditional (e.g., PubMed) sources. Although there is significant existing research examining the relationship between dementia and COVID‐19–related outcomes, this research is primarily outside the United States and minimally examines subtypes of dementia.
2. **Interpretation**: Our findings show that all‐cause dementia is related to an increased in‐hospital mortality risk even when accounting for COVID‐19 vaccination; this relationship is even stronger when using discharge to hospice as an equivalent to mortality, and patients with Alzheimer's disease (AD) and vascular dementia. Patients with all‐cause and AD dementia are less likely to be admitted to the intensive care unit. Patients with all‐cause, AD, and vascular dementia have extended hospital stays compared to control patients.
3. **Future directions**: Future work should examine how dementia severity, code status, provider perception of patients with dementia, and hospitalization policies impact COVID‐19–related outcomes in patients with, versus without, dementia.

### 2.4 Patient characteristics

The following patient characteristics were extracted from the EHR: age, sex, race, health‐care system, number of COVID‐19 vaccinations prior to hospitalization (i.e., 0, 1, 2, or 3 doses), cigarette smoking status, and co‐occurring medical conditions diagnosed at admission, discharge, or encounter (diabetes, chronic obstructive pulmonary disease [COPD], heart disease and heart failure, chronic renal failure, cancer, hyperlipidemia, cirrhosis [see supporting information for ICD‐10 codes]). Co‐occurring medical conditions therefore capture only diagnostic codes associated with the hospital encounter rather than looking back in time for medical history. See Table 1 for patient characteristics. The above characteristics, except for age (i.e., matching variable), were included as a priori covariates in adjusted multivariable models due to their demonstrated impact on the primary outcomes.^49^, ^50^, ^51^, ^52^, ^53^, ^54^, ^55^, ^56^, ^57^

**TABLE 1:** Demographics of dementia and control groups.

	All‐cause dementia	Control	χ^2^	Alzheimer's disease	Control	χ^2^	Vascular dementia	Control	χ^2^
*N*	11,647	11,647	N/A	2648	2648	N/A	1219	1219	N/A
Age (M, SD)	81.29 (8.42)	81.29 (8.42)	N/A	82.21 (7.50)	82.21 (7.50)	N/A	78.14 (9.61)	78.14 (9.61)	N/A
Sex (% female)	56.4	52.6	33.21^***^	61.4	53.0	38.35^***^	50.3	50.9	0.11
Race (%)			64.25^***^			7.05			85.56^***^
Black	19.1	16.0		16.0	15.7		34.1	19.1	
White	69.8	71.0		72.4	72.1		56.2	67.2	
AI/AN	0.2	0.2		0.2	0.3		0.4	0.3	
Asian	2.8	2.8		2.3	2.9		2.1	2.9	
Native Hawaiian or Pacific Islander	0.2	0.3		0.1	0.3		0.1	0.6	
Other race	6.4	8.1		7.4	7.5		5.2	8.9	
> One race	0.3	0.2		0.3	0.2		0.2	0.2	
Unknown/missing	1.2	1.3		1.3	0.9		1.7	0.8	
Health system^a^			1916.66^***^			439.96^***^			343.07^***^
No. COVID‐19 vaccination before hospitalization (%)			91.73^***^			46.128^***^			10.15^*^
0	83.5	79.0		83.9	76.8		83.5	79.2	
1	3.5	3.9		3.5	4.2		3.9	3.7	
2	10.5	13.1		10.1	14.4		10.3	13.5	
3	2.5	4.0		2.6	4.6		2.3	3.5	
Cigarette smoking status			49.66^***^			30.02^***^			23.23^***^
Never	48.0	46.1		50.8	45.0		41.3	44.0	
Former	36.3	40.3		34.4	41.6		41.3	42.6	
Current	5.0	3.9		3.3	3.4		8.8	4.0	
Missing	10.8	9.8		11.5	10.0		9.4	9.4	
Diabetes (% positive)	38.0	31.4	110.85^***^	33.7	30.7	4.13^*^	47.1	34.5	40.28^***^
COPD (% positive)	18.5	17.8	1.67	14.4	18.6	16.58^***^	21.5	19.3	1.84
CAD & CHF (% positive)	34.0	43.9	20.50^***^	39.5	44.1	11.55^***^	52.3	42.7	22.13^***^
Chronic Renal Failure (% positive)	34.0	30.6	31.27^***^	30.1	30.2	0.02	38.5	29.8	20.50^***^
Cancer (% positive)	6.1	9.4	92.85^***^	5.4	9.3	28.93^***^	5.7	8.6	7.54^**^
Cirrhosis (% positive)	1.2	1.3	0.23	0.6	1.1	3.63	1.7	1.1	1.91
Lipid metabolism disorders (% positive)	54.3	48.1	88.82^***^	52.4	49.1	5.85^*^	60.6	48.6	35.28^***^

Abbreviations: χ^2^, chi‐square statistic; AI/AN, American Indian and Alaska Native; COPD, chronic obstructive pulmonary disease; CAD & CHF, coronary artery disease and congestive heart failure.

^a^ Frequencies of each of the 21 anonymized health systems were included in analyses, but not presented in the table for space purposes. Each control group was matched on a 1:1 basis on age and controls had no dementia diagnosis of any kind.

* *p *< 0.05.

** *p *< 0.01.

*** *p *< 0.001.

### 2.5 Statistical analysis

#### 2.5.1 Primary analyses

Matching was conducted on a 1:1 ratio using SAS GMATCH macro^58^ to create samples that were aligned on age while allowing for variability in important predictors used as covariates. Race and sex were not included in the matching process to allow for examination of these factors as potential moderators of the relation between dementia diagnosis and COVID‐19–related severity outcomes. Separate matched samples were created for each of the three dementia groups (all‐cause dementia, AD, and vascular dementia). Independent‐samples *t* tests revealed matching was successful and diagnostic (dementia) versus control (no dementia) groups did not significantly differ by age. Chi‐square analyses were conducted to examine group differences between the diagnostic and control groups (Table 1).

Separate binary logistic regressions were conducted to examine the relations of dementia types (all‐cause dementia, AD, and vascular dementia) on both mortality and ICU admission. After initial unadjusted analyses, relevant covariates (see Patient characteristics section) were included in the models. Due to the positive skew of the distribution of days in the hospital and the count nature of this variable (in days among those who did not die during hospitalization), separate negative binomial regressions^59^ were used to examine the relations of dementia status (all‐cause dementia, AD, and vascular dementia) with hospital stay duration. Hospital duration was rounded up to the nearest integer to allow for these analyses (i.e., both 1.7 and 1.2 days in the hospital would round to 2 days in the hospital). Like other analyses, adjusted regressions accounted for the potential impact of relevant covariates in the model.

#### 2.5.2 Secondary, post hoc, and sensitivity analyses

Secondary analyses first examined the moderating effect of sex (coded as: 0 = female, 1 = male) and race (recoded as: 0 = Black, 1 = White) on unadjusted and adjusted models of dementia types on all outcome variables. Given the increased likelihood of dementia diagnosis for females and Black individuals,^5^ we were interested in whether sex and/or race moderated the effect of all types of dementia on primary outcome variables. Multivariate logistic and negative binomial regressions contrasted each dementia group (all‐cause dementia, AD, vascular dementia) with its control group for each outcome variable (mortality, ICU admission, hospital duration). In moderation analyses involving sex, we tested the effect of sex and other covariates and the added interaction term (sex x dementia diagnosis). In moderation analyses of race, we tested the effect of recoded race and all other covariates and the added interaction term (race x dementia diagnosis). After fitting each model, we computed the average marginal effect of dementia for each subgroup and statistically tested for group differences in the magnitude of this effect to identify and describe significant moderation effects.^60^, ^61^ Post hoc analyses examined whether treating discharge to hospice as a mortality equivalent would affect mortality analyses, due to the unexpected initial mortality findings. Specifically, we created a variable combining discharge to hospice status and mortality in hospital to examine mortality, broadly defined. Any patient who either died in the hospital or was discharged to hospice in any form was labeled as positive for this outcome. We then conducted separate unadjusted and adjusted binary logistic regressions using the same covariates as in initial mortality analyses to examine the impact of all‐cause dementia, AD, and vascular dementia on mortality, broadly defined.

Finally, sensitivity analyses of the link between dementia types and mortality were conducted including all relevant covariates except COVID‐19 vaccination history to examine whether this factor was the main contributor to mortality risk found in the main analyses. A second sensitivity analysis was conducted by limiting analyses to patients and controls who received no COVID‐19 vaccine doses. We also ran primary analyses at different time points throughout the data collection period to ensure results did not substantively change throughout that time.

## 3 RESULTS

### 3.1 Differences between diagnostic and control groups in patient characteristics

Table 1 shows differences between diagnostic and control groups for all types of dementia. The all‐cause dementia group and AD groups had a higher proportion of females than their respective age‐matched control groups, consistent with the increased prevalence of dementia among women in the general population.^5^ All dementia groups differed in race, health‐care system, and smoking status from their respective age‐matched controls. All dementia groups had fewer COVID‐19 vaccinations, higher rates of diabetes, lower cancer rates, and higher rates of lipid metabolism disorders than their respective age‐matched controls. The vascular dementia group showed higher heart disease rates than its control group, while the all‐cause dementia and AD groups had lower rates of heart disease than their respective controls. Vascular and all‐cause dementia groups had higher rates of chronic renal failure than their respective control groups. Only the AD group showed lower rates of COPD than their respective control group.

### 3.2 Associations between dementia and mortality

**FIGURE 1 alz14136-fig-0001:**
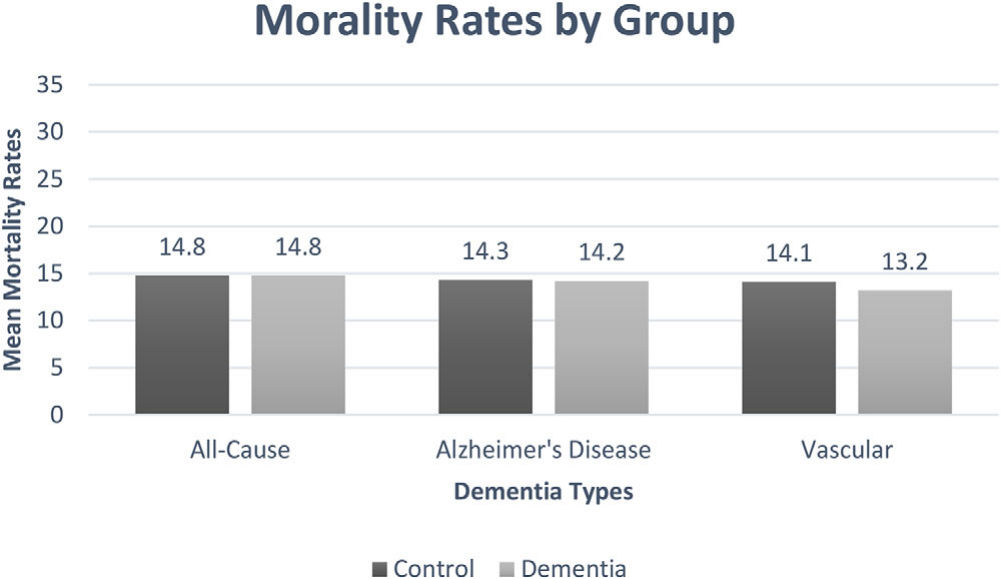
Mortality rates for all‐cause, Alzheimer's disease, and vascular dementia with respective control groups.

Contrary to hypotheses, neither the all‐cause dementia, AD, nor vascular dementia groups (mortality rate = 14.8%, 14.2%, and 13.2%, respectively) differed from their corresponding controls (mortality rate = 14.8%, 14.3%, and 14.1%, respectively) in terms of mortality risk in unadjusted analyses (*P*’s > 0.05). However, adjusted analyses revealed that patients with all‐cause dementia, but not AD or vascular dementia, were at increased risk for mortality (OR = 1.08) compared to patients without dementia. See Table 2 for mortality risk analyses and Figure 1 for mortality comparisons. Full covariate analyses are available in supporting information. Results remained substantively unchanged throughout the study data collection period.

**TABLE 2:** Mortality logistic regression unadjusted and adjusted analyses.

	Unadjusted						Adjusted					
				95% CI						95% CI		
	*B*	*SE*	OR	LL	UL	*p*	*B*	*SE*	OR	LL	UL	*p*
**All dementia**	0.004	0.037	1.004	0.934	1.079	0.912	0.081	0.040	1.084	1.002	1.173	0.045^*^
**Alzheimer's disease**	−0.003	0.079	0.997	0.855	1.163	0.969	0.061	0.087	1.062	0.897	1.259	0.485
**Vascular dementia**	−0.077	0.118	0.926	0.735	1.167	0.517	−0.176	0.135	0.838	0.644	1.092	0.191

*Note*: All dementia: coded as 0 = no dementia, 1 = dementia; Alzheimer's disease: coded as 0 = no dementia of any kind, 1 = Alzheimer's disease; vascular dementia: coded as 0 = no dementia of any kind, 1 = vascular dementia.

Abbreviations: *B*, unstandardized beta; CI, confidence interval; LL, lower limit; OR, odds ratio; *SE*, standard error; UL, upper limit.

* *p *< 0.05.

### 3.3 Post hoc analyses including discharge to hospice as a mortality equivalent

**FIGURE 2 alz14136-fig-0002:**
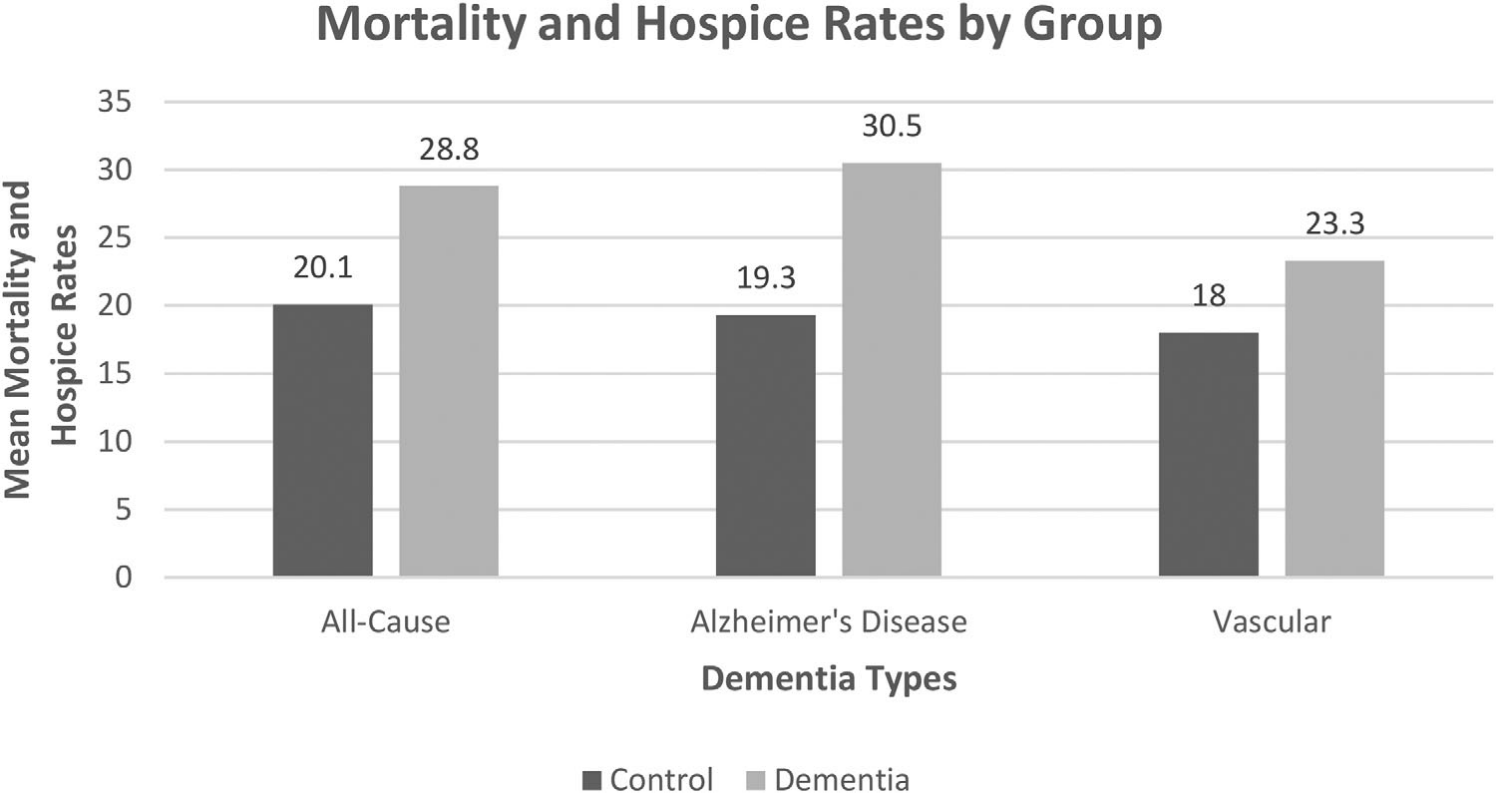
Mortality and discharge to hospice rates for all‐cause, Alzheimer's disease, and vascular dementia with respective control groups.

Analyses of mortality and discharge to hospice (mortality broadly defined) found that patients with all‐cause dementia, AD, and vascular dementia (mortality rate broadly defined = 28.8%, 30.5%, 23.3%, respectively) were more likely to die or be discharged to hospice than patients without any form of dementia (mortality rate broadly defined = 20.1%, 19.3%, 18.0%, respectively; ORs = 1.74, 1.92, and 1.44, respectively). See Table 3 for risk analyses and Figure 2 for mortality broadly defined comparisons. Full covariate analyses are available in supporting information.[Fig alz14136-fig-0001]

**TABLE 3 alz14136-tbl-0003:** Mortality in hospital or discharge to hospice logistic regression unadjusted and adjusted analyses.

				95% CI						95% CI		
	*B*	*SE*	OR	LL	UL	*p*	*B*	*SE*	OR	LL	UL	*p*
**All dementia**	0.478	0.031	1.613	1.519	1.714	<0.001^***^	0.556	0.034	1.744	1.633	1.863	<0.001^***^
**Alzheimer's disease**	0.606	0.065	1.833	1.614	2.082	<0.001^***^	0.655	.071	1.924	1.673	2.213	<0.001^***^
**Vascular dementia**	0.322	0.101	1.379	1.132	1.680	0.001^**^	0.367	0.114	1.443	1.154	1.806	0.001^**^

*Note*: All dementia: coded as 0 = no dementia, 1 = dementia; Alzheimer's disease: coded as 0 = no dementia of any kind, 1 = Alzheimer's disease; vascular dementia: coded as 0 = no dementia of any kind, 1 = vascular dementia.

Abbreviations: *B*, unstandardized beta; CI, confidence interval; LL, lower limit; OR, odds ratio; *SE*, standard error; UL, upper limit.

** *p *< 0.01.

*** *p *< 0.001.

### 3.4 Associations between dementia and ICU admission

Also contrary to hypotheses, patients with all‐cause dementia and AD (percent admitted to ICU = 17.4% and 14.5%, respectively) were less likely to be admitted to the ICU than control patients (percent admitted = 19.3% and 18.5%, respectively) in unadjusted analyses. Patients with vascular dementia (percent admitted = 19.4%) did not differ from controls (percent admitted = 20.5%; Table 4). Adjusted analyses continued to show that patients with all‐cause dementia and AD were less likely to be admitted to the ICU than controls (ORs = 0.89, 0.81, respectively). The relation between vascular dementia and ICU admission remained non‐significant in adjusted analyses. See Table 4 for ICU admission risk analyses. Full covariate analyses are available in the supporting information. Results remained substantively unchanged throughout the study data collection period.

**TABLE 4 alz14136-tbl-0004:** ICU admission logistic regression unadjusted and adjusted analyses.

	Unadjusted						Adjusted					
				95% CI						95% CI		
	*B*	*SE*	OR	LL	UL	*p*	*B*	*SE*	OR	LL	UL	*p*
**All dementia**	−0.127	0.034	0.881	0.824	0.941	<0.001^***^	−0.116	0.037	0.891	0.829	0.957	0.002^**^
**Alzheimer's disease**	−0.292	0.074	0.747	0.646	0.864	<0.001^***^	−0.217	0.081	0.805	0.686	0.943	0.007^*^
**Vascular dementia**	−0.067	0.101	0.925	0.767	1.141	0.510	−0.224	0.116	0.799	0.636	1.004	0.054

*Note*: All dementia: coded as 0 = no dementia, 1 = dementia; Alzheimer's disease: coded as 0 = no dementia of any kind, 1 = Alzheimer's disease; vascular dementia: coded as 0 = no dementia of any kind, 1 = vascular dementia.

Abbreviations: *B*, unstandardized beta; CI, confidence interval; ICU, intensive care unit; LL, lower limit; OR, odds ratio; *SE*, standard error; UL, upper limit.

* *p *< 0.05.

** *p *< 0.01.

*** *p *< 0.001.

### 3.5 Associations between dementia and hospital stay duration

Consistent with hypotheses, unadjusted and adjusted negative binomial regression analyses showed that patients with all types of dementia (all‐cause dementia, AD, vascular dementia) had longer hospital stays than patients without dementia (adjusted incidence rate ratios [IRRs] = 1.23, 1.21, 1.57, respectively). Patients (who did not die while in the hospital) with all‐cause dementia, AD, and vascular dementia spent on average 9.86 (standard deviation [SD] = 12.55), 9.45 (SD = 11.41), and 13.24 (SD = 18.95) days in the hospital, while their respective controls spent 7.95 (SD = 8.92), 7.77 (SD = 7.70),[Table alz14136-tbl-0003], [Fig alz14136-fig-0002] and[Table alz14136-tbl-0004] 7.59[Table alz14136-tbl-0005] (SD = 7.55) days in the hospital. See Table 5 for hospital stay duration analyses. Full covariate analyses are available in supporting information. Results remained substantively unchanged throughout the study data collection period.

**TABLE 5 alz14136-tbl-0005:** Hospital stay duration negative binomial regression unadjusted and adjusted analyses.

	Unadjusted						Adjusted					
				95% CI						95% CI		
	*B*	*SE*	IRR	LL	UL	*p*	*B*	*SE*	IRR	LL	UL	*p*
**All dementia**	0.207	0.0149	1.230	1.195	1.267	0.000^***^	0.203	0.0157	1.225	1.188	1.264	0.000^***^
**Alzheimer's disease**	0.189	0.0313	1.209	1.137	1.285	<0.001^***^	0.193	0.0335	1.213	1.135	1.295	<0.001^***^
**Vascular dementia**	0.531	0.0457	1.701	1.556	1.861	0.000^***^	0.451	0.0518	1.570	1.418	1.738	<0.001^***^

*Note*: All dementia: coded as 0 = no dementia, 1 = dementia; Alzheimer's disease: coded as 0 = no dementia of any kind, 1 = Alzheimer's disease; vascular dementia: coded as 0 = no dementia of any kind, 1 = vascular dementia.

Abbreviations: *B*, unstandardized beta; CI, confidence interval; IRR, incidence rate ratio; LL, lower limit; UL, upper limit.

*** *p *< 0.001.

### 3.6 Are the dementia–outcome associations moderated by sex or race?

Moderation analyses revealed that neither sex nor race moderated the relation between any type of dementia diagnosis and mortality risk (*p*’s > 0.05). Similarly, sex did not moderate the relation between any type of dementia diagnosis and ICU admission risk (*p*’s > 0.05).

The race x vascular dementia product term was significant (*p* = 0.043) in the model testing the relation between vascular dementia and ICU admission status. On average, the marginal effect of vascular dementia increased the probability of ICU admission by 2.1 percentage points (from 21.0% to 23.1%) among Black patients but decreased the probability among White patients by 5.3 percentage points (from 21.3% to 16.0%). A formal test of the group difference in the size of these marginal effects bordered on statistical significance (*p* = 0.053). Race did not moderate the relation between all‐cause dementia or AD and ICU admission risk (*p*’s > 0.05).

For each type of diagnosis, the average marginal effect of dementia on length of hospital stay was significantly larger in men compared to women (*p*’s < 0.006). Among women, the marginal effect of all‐cause dementia was to increase the length of stay by 1.5 days (from 8.3 to 9.8) while in males all‐cause dementia lengthened the stay on average by 2.5 days (from 8.7 to 11.1). In women, the average marginal effect of AD was to increase time in the hospital by 1.3 days (from 8.1 to 9.4) whereas the comparable effect in men was an increase of 2.4 days (from 8.4 to 10.9). Vascular dementia on average increased time in the hospital by 3.4 days among women (from 8.6 to 12.0) and 6.3 days (from 8.2 to 14.5) among men.

The average marginal effect of vascular dementia among Black patients was an increased length of stay of 6.3 days (from 8.6 to 14.9). Among White patients, vascular dementia increased length of stay on average by 4.1 days (from 8.1 to 12.2). The group difference in the magnitude of the average marginal effect was significant (*p* = 0.018). Race did not moderate relations between all‐cause dementia or AD and hospital stay duration. Figures S3–S7 in supporting information show plots of significant interaction effects.

### 3.7 Are the dementia–outcome associations explained by COVID‐19 vaccination?

Sensitivity analyses of the impact of all‐cause, AD, and vascular dementia on mortality without the inclusion of the COVID‐19 vaccination history variable found that results remained substantively unchanged from primary analyses. Compared to patients without dementia, patients with all‐cause dementia were at increased risk of mortality (OR = 1.11), but there was no difference in risk of mortality for patients with AD or vascular dementia (*p*’s > 0.05).

A second set of sensitivity analyses of the impact of all‐cause, AD, and vascular dementia on mortality limited to participants with zero doses of COVID‐19 vaccines prior to admission found that the association between all‐cause dementia and mortality became non‐significant, and there remained no difference in mortality risk for patients with AD or vascular dementia compared to those without any dementia diagnosis (*p*’s > 0.05).

## 4 DISCUSSION

This study examined the impact of all‐cause dementia and the two most common forms of dementia (AD and vascular dementia) on COVID‐19–related outcomes of in‐hospital mortality, ICU admission status, and hospital stay duration. Partially consistent with hypotheses, after accounting for relevant covariates, all‐cause dementia, but not AD or vascular dementia, was associated with an increased risk of mortality. ICU admissions were less likely among patients with all‐cause dementia and AD compared to controls in adjusted analyses; patients with vascular dementia did not differ from controls in terms of ICU risk when accounting for relevant covariates. Consistent with hypotheses, among patients discharged alive, those with all‐cause dementia, AD, and vascular dementia had longer hospital stays than patients without dementia.

When accounting for comorbid medical conditions, smoking status, demographic factors, and COVID‐19 vaccinations prior to admission, this large retrospective study of 21 hospital systems in the United States found patients with all‐cause dementia were 1.08 times more likely to die in the hospital than patients without any dementia diagnosis. This finding is consistent with almost all existing research, both within and outside the United States.^7^, ^8^ However, the increased risk of mortality associated with all‐cause dementia is much lower than reported in previous studies. Additionally, previous research found that patients with AD were also at increased risk for mortality,^34^ while our study did not. We believed the difference in risk for all‐cause dementia may be related to the timing of previous studies, as all but two^13^, ^34^ were conducted prior to the release of the first COVID‐19 vaccine in late 2020.^62^ However, the 8% increased risk of mortality found in our study is lower than the 33% increased risk found in the most recent study conducted among 50 hospitals in Germany that similarly matched patients by age, in addition to sex, race, and comorbid conditions.^13^ Another key difference between the current study and previous research is that ours is the only study, known to the authors, that accounted for COVID‐19 vaccination. In fact, COVID‐19 vaccination history significantly impacted all COVID‐19–related outcomes among all examined subtypes of dementia such that patients with some vaccinations had lower rates of mortality, lower ICU admissions, and shorter hospital stays.

Although these study differences appear to suggest that the inclusion of COVID‐19 vaccination prior to admission in the model may attenuate the relation between dementia and mortality, sensitivity analyses removing COVID‐19 vaccination (but retaining all other covariates) only resulted in a 2% increased risk for all‐cause dementia and did not result in a significant association between AD or vascular dementia and mortality in the hospital. Additional sensitivity analyses limiting analyses to only patients who had no COVID‐19 vaccination found similar results, but the relation between all‐cause dementia and mortality became non‐significant. Therefore, COVID‐19 vaccination did not account for the unexpected results in this study.

Conversely, examination of discharge to hospice as a proxy for mortality indicated that patients with all‐cause, AD, and vascular dementia all showed a much stronger increased risk of mortality broadly defined than patients without dementia. Therefore, the lower‐than‐expected mortality findings may be a function of documentation of death within the hospital system, rather than a different finding in terms of disease pathology. That is to say that when accounting for discharge as a proxy for death, we found that patients with dementia are as, if not more, likely to have negative health outcomes than patients within and outside the United States. Further, the highest increased risk (92%) was found among patients with AD, rather than all‐cause dementia. This is surprising as AD tends to progress more slowly than other forms of dementia.^63^ However, diagnosis of AD dementia requires a relatively intense diagnostic process, including more thorough neuropsychological testing and blood work, in addition to magnetic resonance imaging brain scans.^64^ Therefore, patients with AD may have advanced to a more severe disease state before being diagnosed than patients with other forms of dementia.

Findings of ICU admission were unexpected in that patients with all‐cause and AD dementia were less likely to be admitted for more advanced care. This is consistent with a previous study conducted in the United States;^37^ however, the previous study did not differentiate by subtype of dementia. The reason patients with dementia may have been less likely to be admitted to the ICU is the clinician perception and/or bias of the patients’ overall health and belief they could not tolerate aggressive medical treatments within the ICU. Research has shown that patients with a dementia diagnosis are less likely to receive intensive life‐saving care,^65^ and this is further complicated by do‐not‐resuscitate orders that become more common as people age.^66^ Interestingly, there was no difference between patients with and without vascular dementia in terms of ICU admission. Additionally, race moderated the relation between vascular dementia and ICU admission such that Black individuals with vascular dementia had increased rates of ICU admission while White patients with vascular dementia had decreased rates of admission. This may be related to the elevated rates and increased severity of medical comorbidities associated with vascular disease in Black individuals^67^, ^68^, ^69^ that required more intensive care and/or to the lower rates of do not resuscitate orders in White versus Black individuals.^70^

As expected, the duration of hospital stay was longer among patients with all‐cause dementia, AD, and vascular dementia compared to patients without dementia. Patients with all‐cause, AD, and vascular dementia were 21%, 23%, and 54% more likely to spend another day in the hospital than patients without dementia, holding all other variables constant (see the Associations between dementia and hospital stay duration section). Findings are consistent with research done in the Untied States showing dementia in the context of COVID‐19 was associated with increased length of hospital stay,^37^ but the previous work did not analyze by dementia subtype.^71^ Moderation analyses showed that men with an all‐cause, AD, or vascular dementia diagnosis were particularly impacted by their diagnosis in terms of increasing their hospitalization duration. Race also moderated the effect of vascular dementia on hospital stay such that Black adults with dementia showed greater increases in length of stay than White patients with vascular dementia. Again, this may relate to the elevated rates and increased severity of medical comorbidities associated with vascular disease in Black individuals.^67^, ^68^, ^69^ However, this is contrary to recent research among Medicaid beneficiaries showing that White adults with dementia, but less so Black adults with dementia, show higher hospitalization rates.^71^ While the Medicaid‐based study^71^ used all‐cause dementia as their main grouping, we only found a moderate effect in vascular dementia (not all‐cause or AD dementia). However, this study was not exclusive to Medicaid beneficiaries, who must have limited income to qualify for such services and may therefore represent a greater breadth in socioeconomic status. Overall, the greatest increase in hospital stay was found in patients with vascular dementia. This could again highlight the increased medical comorbidities occurring in patients with vascular dementia compared to other forms of dementia.^72^

This study has certain strengths and limitations worth noting. Strengths of this work include the diversity of the sample, large representation of various forms of dementia in the group, extended timeline of data collection through multiple waves of the COVID‐19 pandemic, inclusion of COVID‐19 vaccination as a covariate, and representativeness across multiple regions in the country while controlling for the impact of recruitment location (via health‐care site) on findings. Most notably, the inclusion of post hoc analyses examining the impact of a combined variable of in‐hospital mortality and discharge to hospice to represent mortality, broadly defined, is a strength of this study compared to previous literature. Given how much the inclusion of discharge to hospice impacted mortality results, findings suggest that this technique should be used for a more robust picture of how dementia relates to mortality. Limitations include the lack of information on dementia severity and status of do‐not‐resuscitate orders as these may have impacted mortality outcomes and use of intensive care measures. Another limitation that all EHR data studies are subject to is missingness of data. Specifically, participants in this study may not have returned to the tested health‐care system for follow‐up, resulting in missing data. In an effort to minimize this impact and ensure some history of comorbid conditions, one of the study criteria was that participants needed to have had a previous attended appointment within the health‐care system. Similarly, dementia is commonly undetected and underdiagnosed,^73^ and this study, like all other EHR studies for dementia, may have underestimated the number of participants with dementia. We also did not have access to socioeconomic factors that could have confounded results. Additionally, authors chose to match participants on only age, and no other relevant factors, to allow for examination of relevant covariates as potential moderators of the relation between dementia and COVID‐19 related outcomes. While Kostev et al.^13^ found that not matching on all factors inflated the impact of dementia on mortality, our study showed much lower associations than reported elsewhere, except when accounting for post‐discharge hospice care as a mortality equivalent.

Overall, this study of hospitalized patients with COVID‐19 found a lower‐than‐expected increased risk of mortality for patients with all‐cause dementia, but this risk was greatly increased when including discharge to hospice as a mortality equivalent. Further, patients with AD and vascular dementia showed a similar increased risk when accounting for discharge to hospice. ICU admission risk was lower among patients with all‐cause and AD dementia, highlighting that patients with dementia receive less intensive lifesaving care. Finally, hospital stays were longer for patients with all‐cause, AD, and vascular dementia highlighting the clinical implications of dementia in the context of COVID‐19. Future work should examine how dementia severity, code status, provider perception of patients with dementia, and hospitalization policies impact COVID‐19–related outcomes in patients with, versus without, dementia. Moreover, future work should examine potential biological and societal mechanisms that result in different hospital outcomes for different types of dementia.

## CONFLICT OF INTEREST STATEMENT

A.L.J. holds leadership positions for the Society for Research on Nicotine and Tobacco and the Wisconsin African American Tobacco Prevention Network. T.B.B. occupies the Glaxo‐Wellcome Chair in the University of Wisconsin School of Medicine Department of Medicine. N.C. receives consulting fees from NewAmsterdam Pharmaceutical Inc. and is a volunteer member of the Medical and Science Board for the Wisconsin Alzheimer's Association and the Alzheimer's Foundation of America. K.C., W.S., and M.C.F. have no conflicts of interest to report. Author disclosures are available in the supporting information.

## CONSENT STATEMENT

The University of Wisconsin‐Madison Health Sciences Minimal Risk Institutional Review Board determined that the study met the criteria for a human subjects research exemption and qualified for a waiver of informed consent under the Federal Common Rule. Therefore, consent was not required for human subjects.

## Supporting information

Supporting Information

Supporting Information
