# Supplementary material for: COVID‐19 outcomes among patients with dementia and age‐matched controls who were hospitalized in 21 US health‐care systems
Source: Alzheimers Dement. 2024 Jul 29;20(9):6395–406. doi: 10.1002/alz.14136 (PMC11497724; doi:10.1002/alz.14136)
Supplement: Supplementary file 1 — Supporting Information [file ALZ-20-6395-s001.pdf]

Supplemental Materials

for

COVID-19 Outcomes Among Patients with Dementia and Age-Matched Controls who were Hospitalized in 21 U.S. Healthcare Systems

Contents

Text S1.	Distributions of all-cause dementia subtypes.
Text S2.	ICD-10 codes of comorbid medical conditions.
Text S3.	Mortality Logistic Regression Unadjusted and Adjusted Analyses
Text S4.	Mortality in Hospital or Discharge to Hospice Logistic Regression Unadjusted and Adjusted Analyses
Text S5.	ICU Admission Logistic Regression Unadjusted and Adjusted Analyses
Text S6.	Hospital Stay Duration Negative Binomial Regression Unadjusted and Adjusted Analyses
Figure S1.	Map of Health Systems.
Figure S2.	Distribution of Dementia Subtypes by Health System.
Figure S3.	Race by Vascular Dementia Interaction for ICU Admission.
Figure S4.	Sex by All-Cause Dementia Interaction for Hospital Stay Duration.
Figure S5.	Sex by Alzheimer's Disease Interaction for Hospital Stay Duration.
Figure S6.	Sex by Vascular Dementia Interaction for Hospital Stay Duration.
Figure S7.	Race by Vascular Dementia Interaction for Hospital Stay Duration.

Text S1. Distributions of all-cause dementia subtypes.

ICD-10 codes and associated frequency for all subtypes of dementia used to create the all-cause dementia variable. Participants could have more than one subtype of dementia, which is why the total number of patients is above the total of all-cause dementia patients (N = 11,647).

ICD-10 code/Description	Frequency
F01/Vascular Dementia	1219
F02/Dementia in Other Diseases	3667
F03/Unspecified Dementia	7537
G30/Alzheimer's Disease	2648
G31.0/Frontotemporal Dementia	80
G31.01/Picks Disease Dementia	13
G31.09/Other Frontotemporal Dementia	70
G31.1/Senile Degeneration of Brain	3
G31.83/Dementia with Lewy Bodies	227

Text S2. ICD-10 codes of comorbid medical conditions

ICD-10 codes for the following medical conditions were extracted from the admission, encounter, and discharge diagnosis fields in the electronic health record and were combined into a composite diagnosis when necessary.

Comorbid disorder	ICD-10 codes
Diabetes	E08, E09, E10, E11, E13
Chronic Obstructive Pulmonary Disease (COPD)	J43, J44
Heart disease and heart failure	I25, I24, I50
Chronic renal failure	N18.x, Z99.2
Cancer	C00 – C95
Hyperlipidemia	K78.1 K78.2 K78.4 K78.5
Cirrhosis	K70.31, K74.x

Text S3. Mortality Logistic Regression Unadjusted and Adjusted Analyses

	Unadjusted						*Adjusted*					
	B	***SE***	**OR**	**95% CI**		***p***	***B***	***SE***	**OR**	**95% CI**		***p***
				**LL**	**UL**					**LL**	**UL**	
**All Dementia**	.004	.037	1.004	.934	1.079	.912	.081	.040	1.084	1.002	1.173	.045*
Sex	---	---	---	---	---	---	.300	.039	1.349	1.250	1.456	<.001***
Race[a]	---	---	---	---	---	---						.010*
Health System[a]	---	---	---	---	---	---						<.001***
COVID-19 vaccination	---	---	---	---	---	---						<.001***
0 vs. 1	---	---	---	---	---	---	-.687	.121	.503	.397	.638	<.001***
0 vs. 2	---	---	---	---	---	---	-.718	.072	.488	.423	.562	<.001***
0 vs. 3	---	---	---	---	---	---	-.987	.148	.373	.279	.498	<.001***
Smoking	---	---	---	---	---	---						.010*
Never vs. former	---	---	---	---	---	---	.050	.044	1.052	.965	1.146	.250
Never vs. current	---	---	---	---	---	---	-.297	.106	.743	.604	.915	.005**
Never vs. missing	---	---	---	---	---	---	-.020	.068	.980	.858	1.120	.770
Diabetes	---	---	---	---	---	---	.194	.042	1.215	1.119	1.319	<.001***
COPD	---	---	---	---	---	---	.023	.052	1.024	.925	1.133	.651
CAD & CHF	---	---	---	---	---	---	.386	.042	1.471	1.355	1.597	<.001***
Chronic Renal Failure	---	---	---	---	---	---	.327	.042	1.387	1.277	1.508	<.001***
Cancer	---	---	---	---	---	---	.253	.069	1.288	1.124	1.475	<.001***
Cirrhosis	---	---	---	---	---	---	.278	.156	1.320	.972	1.792	.075
Lipid Metabolism Disorders	---	---	---	---	---	---	-.175	.042	.839	.772	.912	<.001***
**Alzheimer's Disease**	-.003	.079	.997	.855	1.163	.969	.061	.087	1.062	.897	1.259	.485
Sex	---	---	---	---	---	---	.324	.084	1.383	1.173	1.631	<.001***
Race[a]	---	---	---	---	---	---						.007**
Health System[a]	---	---	---	---	---	---						<.001***
COVID-19 vaccination	---	---	---	---	---	---						<.001***
0 vs. 1	---	---	---	---	---	---	-1.019	.293	.361	.203	.641	<.001***
0 vs. 2	---	---	---	---	---	---	-.973	.163	.378	.275	.520	<.001***
0 vs. 3	---	---	---	---	---	---	-1.288	.333	.276	.144	.530	<.001***

Smoking	---	---	---	---	---	---						.392
Never vs. former	---	---	---	---	---	---	.001	.094	1.001	.832	1.204	.991
Never vs. current	---	---	---	---	---	---	-.446	.264	.640	.381	1.074	.091
Never vs. missing	---	---	---	---	---	---	-.037	.142	.964	.729	1.274	.797
Diabetes	---	---	---	---	---	---	.124	.091	1.132	.948	1.352	.170
COPD	---	---	---	---	---	---	.097	.114	1.102	.881	1.379	.394
CAD & CHF	---	---	---	---	---	---	.281	.090	1.324	1.109	1.581	.002**
Chronic Renal Failure	---	---	---	---	---	---	.375	.091	1.455	1.217	1.739	<.001***
Cancer	---	---	---	---	---	---	.319	.150	1.376	1.025	1.848	.034*
Cirrhosis	---	---	---	---	---	---	-.425	.535	.654	.229	1.865	.427
Lipid Metabolism Disorders	---	---	---	---	---	---	.019	.092	1.019	.852	1.220	.833
**Vascular Dementia**	-.077	.118	.926	.735	1.167	.517	-.176	.135	.838	.644	1.092	.191
Sex	---	---	---	---	---	---	.145	.125	1.157	.906	1.477	.243
Race[a]	---	---	---	---	---	---						.493
Health System[a]	---	---	---	---	---	---						.430
COVID-19 vaccination	---	---	---	---	---	---						<.001***
0 vs. 1	---	---	---	---	---	---	-.863	.405	.422	.191	.932	.033*
0 vs. 2	---	---	---	---	---	---	-1.167	.269	.311	.184	.528	<.001***
0 vs. 3	---	---	---	---	---	---	-.968	.476	.380	.149	.966	.042*
Smoking	---	---	---	---	---	---						.013*
Never vs. former	---	---	---	---	---	---	.001	.141	1.001	.759	1.321	.993
Never vs. current	---	---	---	---	---	---	-.521	.309	.594	.324	1.088	.092
Never vs. missing	---	---	---	---	---	---	.553	.221	1.739	1.128	2.681	.012*
Diabetes	---	---	---	---	---	---	.289	.133	1.335	1.028	1.732	.030*
COPD	---	---	---	---	---	---	.188	.156	1.207	.889	1.639	.228
CAD & CHF	---	---	---	---	---	---	.498	.137	1.645	1.257	2.154	<.001***
Chronic Renal Failure	---	---	---	---	---	---	.072	.136	1.075	.823	1.404	.596
Cancer	---	---	---	---	---	---	.116	.243	1.123	.698	1.807	.634
Cirrhosis	---	---	---	---	---	---	.383	.468	1.467	.587	3.670	.412
Lipid Metabolism Disorders	---	---	---	---	---	---	-.190	.139	.827	.629	1.086	.172

* $p < .05$, ** $p < .01$, *** $p < .001$

Note. *B* = Unstandardized Beta; *SE* = Standard Error; OR = Odds Ratio; 95% CI = 95% Confidence Interval; LL = Lower Limit; UL = Upper Limit; All Dementia: coded as 0 = no dementia, 1 = dementia; Alzheimer's Disease: coded as 0 = no dementia of any kind, 1 = Alzheimer's disease; Vascular Dementia: coded as 0 = no dementia of any kind, 1 = Vascular Dementia. COPD = chronic obstructive pulmonary disease; CAD & CHF = coronary artery disease and congestive heart failure. [a]Race and Health System were each included as categorical variables in analyses, but presentation of the comparisons of subcategories was not included in tables for space purposes. These results can be obtained from the author by request.

Text S4. Mortality in Hospital or Discharge to Hospice Logistic Regression Unadjusted and Adjusted Analyses

	B	*SE*	OR	95% CI		*p*	*B*	*SE*	OR	95% CI		*p*
				LL	UL					LL	UL	
**All Dementia**	.478	.031	1.613	1.519	1.714	<.001***	.556	.034	1.744	1.633	1.863	<.001***
Sex	---	---	---	---	---	---	.175	.032	1.191	1.118	1.269	<.001***
Race[a]	---	---	---	---	---	---						<.001***
Health System[a]	---	---	---	---	---	---						<.001***
COVID-19 vaccination	---	---	---	---	---	---						<.001***
0 vs. 1	---	---	---	---	---	---	-.637	.095	.529	.439	.637	<.001***
0 vs. 2	---	---	---	---	---	---	-.671	.057	.511	.457	.571	<.001***
0 vs. 3	---	---	---	---	---	---	-.831	.110	.436	.351	.541	<.001***
Smoking	---	---	---	---	---	---						<.001***
Never vs. former	---	---	---	---	---	---	-.023	.036	.712	.601	.843	.519
Never vs. current	---	---	---	---	---	---	-.340	.086	1.211	1.087	1.349	<.001***
Never vs. missing	---	---	---	---	---	---	.191	.055	1.031	.963	1.104	<.001***
Diabetes	---	---	---	---	---	---	.030	.035	1.040	.956	1.132	.386
COPD	---	---	---	---	---	---	.040	.043	1.427	1.333	1.526	.357
CAD & CHF	---	---	---	---	---	---	.355	.034	1.378	1.286	1.477	<.001***
Chronic Renal Failure	---	---	---	---	---	---	.321	.035	1.420	1.267	1.591	<.001***
Cancer	---	---	---	---	---	---	.351	.058	1.179	.899	1.546	<.001***
Cirrhosis	---	---	---	---	---	---	.165	.138	.840	.784	.899	.233
Lipid Metabolism Disorders	---	---	---	---	---	---	-.175	.035	.712	.601	.843	<.001***
**Alzheimer's Disease**	.606	.065	1.833	1.614	2.082	<.001***	.655	.071	1.924	1.673	2.213	<.001***
Sex	---	---	---	---	---	---	.252	.069	1.286	1.124	1.472	<.001***
Race[a]	---	---	---	---	---	---						.019*
Health System[a]	---	---	---	---	---	---						.004**
COVID-19 vaccination	---	---	---	---	---	---						<.001***
0 vs. 1	---	---	---	---	---	---	-.829	.210	.436	.289	.659	<.001***
0 vs. 2	---	---	---	---	---	---	-.770	.120	.463	.366	.586	<.001***
0 vs. 3	---	---	---	---	---	---	-.872	.223	.418	.270	.648	<.001***
Smoking	---	---	---	---	---	---						.002**

Never vs. former	---	---	---	---	---	---	-.080	.077	.923	.794	1.073	.299
Never vs. current	---	---	---	---	---	---	-.495	.212	.610	.403	.923	.019*
Never vs. missing	---	---	---	---	---	---	.281	.113	1.324	1.061	1.652	.013*
Diabetes	---	---	---	---	---	---	-.037	.075	.964	.833	1.116	.622
COPD	---	---	---	---	---	---	.014	.095	1.014	.841	1.221	.887
CAD & CHF	---	---	---	---	---	---	.243	.073	1.275	1.105	1.471	<.001***
Chronic Renal Failure	---	---	---	---	---	---	.332	.075	1.394	1.204	1.615	<.001***
Cancer	---	---	---	---	---	---	.415	.125	1.515	1.185	1.937	<.001***
Cirrhosis	---	---	---	---	---	---	-.595	.449	.551	.229	1.329	.185
Lipid Metabolism Disorders	---	---	---	---	---	---	-.070	.073	.932	.808	1.076	.335
**Vascular Dementia**	.322	.101	1.379	1.132	1.680	.001**	.367	.114	1.443	1.154	1.806	.001**
Sex	---	---	---	---	---	---	.093	.106	1.098	.892	1.350	.378
Race[a]	---	---	---	---	---	---						.713
Health System[a]	---	---	---	---	---	---						.527
COVID-19 vaccination	---	---	---	---	---	---						<.001***
0 vs. 1	---	---	---	---	---	---	-.693	.320	.500	.267	.936	.030*
0 vs. 2	---	---	---	---	---	---	-.741	.191	.476	.328	.692	<.001***
0 vs. 3	---	---	---	---	---	---	-.737	.367	.479	.233	.983	.045*
Smoking	---	---	---	---	---	---						<.001***
Never vs. former	---	---	---	---	---	---	-.124	.119	.884	.700	1.116	.299
Never vs. current	---	---	---	---	---	---	-.726	.263	.484	.289	.810	.006**
Never vs. missing	---	---	---	---	---	---	.581	.190	1.788	1.232	2.596	.002**
Diabetes	---	---	---	---	---	---	.062	.113	1.064	.853	1.327	.584
COPD	---	---	---	---	---	---	.120	.134	1.128	.867	1.466	.370
CAD & CHF	---	---	---	---	---	---	.450	.115	1.569	1.252	1.966	<.001***
Chronic Renal Failure	---	---	---	---	---	---	.197	.115	1.218	.973	1.524	.086
Cancer	---	---	---	---	---	---	.186	.204	1.204	.807	1.797	.364
Cirrhosis	---	---	---	---	---	---	.054	.439	1.056	.447	2.494	.902
Lipid Metabolism Disorders	---	---	---	---	---	---	-.142	.117	.868	.690	1.091	.225

* $p < .05$, ** $p < .01$, *** $p < .001$

Note. *B* = Unstandardized Beta; *SE* = Standard Error; OR = Odds Ratio; 95% CI = 95% Confidence Interval; LL = Lower Limit; UL = Upper Limit; All Dementia: coded as 0 = no dementia, 1 = dementia; Alzheimer's Disease: coded as 0 = no dementia of any kind, 1 =

Alzheimer's disease; Vascular Dementia: coded as 0 = no dementia of any kind, 1 = Vascular Dementia. COPD = chronic obstructive pulmonary disease; CAD & CHF = coronary artery disease and congestive heart failure. [a]Race and Health System were each included as categorical variables in analyses, but presentation of the comparisons of subcategories was not included in tables for space purposes. These results can be obtained from the author by request.

Text S5. ICU Admission Logistic Regression Unadjusted and Adjusted Analyses

	Unadjusted						***Adjusted***					
	B	***SE***	**OR**	**95% CI**		***p***	***B***	***SE***	**OR**	**95% CI**		***p***
				**LL**	**UL**					**LL**	**UL**	
**All Dementia**	-.127	.034	.881	.824	.941	<.001***	-.116	.037	.891	.829	.957	.002**
Sex	---	---	---	---	---	---	.235	.036	1.265	1.179	1.356	<.001***
Race[a]	---	---	---	---	---	---						<.001***
Health System[a]	---	---	---	---	---	---						<.001***
COVID-19 vaccination	---	---	---	---	---	---						<.001***
0 vs. 1	---	---	---	---	---	---	-.358	.101	.699	.574	.852	<.001***
0 vs. 2	---	---	---	---	---	---	-.401	.059	.670	.597	.752	<.001***
0 vs. 3	---	---	---	---	---	---	-.325	.106	.722	.587	.888	.002**
Smoking	---	---	---	---	---	---						<.001***
Never vs. former	---	---	---	---	---	---	.037	.040	1.038	.959	1.122	.357
Never vs. current	---	---	---	---	---	---	.064	.086	1.066	.901	1.261	.456
Never vs. missing	---	---	---	---	---	---	.259	.063	1.296	1.145	1.467	<.001***
Diabetes	---	---	---	---	---	---	.300	.038	1.349	1.252	1.454	<.001***
COPD	---	---	---	---	---	---	.126	.047	1.135	1.036	1.243	.007**
CAD & CHF	---	---	---	---	---	---	.297	.038	1.346	1.249	1.450	<.001***
Chronic Renal Failure	---	---	---	---	---	---	.099	.039	1.104	1.023	1.192	.011*
Cancer	---	---	---	---	---	---	.146	.063	1.157	1.022	1.310	.021*
Cirrhosis	---	---	---	---	---	---	.257	.143	1.293	.976	1.712	.074
Lipid Metabolism Disorders	---	---	---	---	---	---	-.137	.039	.872	.808	.941	<.001***
**Alzheimer's Disease**	-.292	.074	.747	.646	.864	<.001***	-.217	.081	.805	.686	.943	.007*
Sex	---	---	---	---	---	---	.320	.079	1.377	1.180	1.608	<.001***
Race[a]	---	---	---	---	---	---						.903
Health System[a]	---	---	---	---	---	---						<.001***
COVID-19 vaccination	---	---	---	---	---	---						<.001***
0 vs. 1	---	---	---	---	---	---	-.391	.222	.676	.438	1.045	.078
0 vs. 2	---	---	---	---	---	---	-.578	.134	.561	.431	.730	<.001***
0 vs. 3	---	---	---	---	---	---	-.297	.212	.743	.490	1.126	.162

Smoking	---	---	---	---	---	---						.392
Never vs. former	---	---	---	---	---	---	-.029	.088	.971	.817	1.154	.739
Never vs. current	---	---	---	---	---	---	-.014	.214	.987	.648	1.501	.949
Never vs. missing	---	---	---	---	---	---	.213	.136	1.237	.947	1.616	.119
Diabetes	---	---	---	---	---	---	.282	.085	1.326	1.122	1.567	<.001***
COPD	---	---	---	---	---	---	.039	.108	1.040	.842	1.284	.715
CAD & CHF	---	---	---	---	---	---	.306	.085	1.358	1.150	1.604	<.001***
Chronic Renal Failure	---	---	---	---	---	---	.040	.088	1.040	.876	1.235	.651
Cancer	---	---	---	---	---	---	.279	.139	1.322	1.007	1.735	.044*
Cirrhosis	---	---	---	---	---	---	-.722	.536	.486	.170	1.389	.178
Lipid Metabolism Disorders	---	---	---	---	---	---	-.045	.086	.956	.807	1.131	.599
**Vascular Dementia**	-.067	.101	.925	.767	1.141	.510	-.224	.116	.799	.636	1.004	.054
Sex	---	---	---	---	---	---	.226	.108	1.254	1.016	1.548	.035*
Race[a]	---	---	---	---	---	---						.463
Health System[a]	---	---	---	---	---	---						.001**
COVID-19 vaccination	---	---	---	---	---	---						.006**
0 vs. 1	---	---	---	---	---	---	-.294	.289	.745	.423	1.314	.310
0 vs. 2	---	---	---	---	---	---	-.663	.194	.515	.352	.753	<.001***
0 vs. 3	---	---	---	---	---	---	-.181	.315	.835	.450	1.548	.567
Smoking	---	---	---	---	---	---						<.001***
Never vs. former	---	---	---	---	---	---	-.078	.123	.925	.727	1.176	.524
Never vs. current	---	---	---	---	---	---	.474	.210	1.606	1.063	2.427	.024*
Never vs. missing	---	---	---	---	---	---	.613	.199	1.846	1.249	2.727	.002**
Diabetes	---	---	---	---	---	---	.317	.116	1.373	1.095	1.722	.006**
COPD	---	---	---	---	---	---	-.059	.139	.943	.718	1.239	.674
CAD & CHF	---	---	---	---	---	---	.444	.117	1.560	1.239	1.964	<.001***
Chronic Renal Failure	---	---	---	---	---	---	.032	.118	1.032	.819	1.302	.787
Cancer	---	---	---	---	---	---	.137	.203	1.146	.771	1.705	.500
Cirrhosis	---	---	---	---	---	---	.029	.442	1.029	.433	2.445	.948
Lipid Metabolism Disorders	---	---	---	---	---	---	.003	.121	1.003	.792	1.271	.979

* $p < .05$, ** $p < .01$, *** $p < .001$

Note. *B* = Unstandardized Beta; *SE* = Standard Error; OR = Odds Ratio; 95% CI = 95% Confidence Interval; LL = Lower Limit; UL = Upper Limit; All Dementia: coded as 0 = no dementia, 1 = dementia; Alzheimer's Disease: coded as 0 = no dementia of any kind, 1 = Alzheimer's disease; Vascular Dementia: coded as 0 = no dementia of any kind, 1 = Vascular Dementia. COPD = chronic obstructive pulmonary disease; CAD & CHF = coronary artery disease and congestive heart failure. [a]Race and Health System were each included as categorical variables in analyses, but presentation of the comparisons of subcategories was not included in tables for space purposes. These results can be obtained from the author by request.

Text S6. Hospital Stay Duration Negative Binomial Regression Unadjusted and Adjusted Analyses

	Unadjusted						*Adjusted*					
	B	***SE***	**IRR**	**95% CI**		***p***	***B***	***SE***	**IRR**	**95% CI**		***p***
				**LL**	**UL**					**LL**	**UL**	
**All Dementia**	.207	.0149	1.230	1.195	1.267	.000***	.203	.0157	1.225	1.188	1.264	.000***
Sex	---	---	---	---	---	---	.089	.0155	1.093	1.060	1.127	<.001***
Race[a]	---	---	---	---	---	---						
Health System[a]	---	---	---	---	---	---						
COVID-19 vaccination	---	---	---	---	---	---						
0 vs. 1	---	---	---	---	---	---	-.118	.0389	.888	.823	.959	.002**
0 vs. 2	---	---	---	---	---	---	-.098	.0231	.907	.867	.949	<.001***
0 vs. 3	---	---	---	---	---	---	-.224	.0412	.799	.737	.866	<.001***
Smoking	---	---	---	---	---	---						
Never vs. former	---	---	---	---	---	---	-.012	.0173	.988	.955	1.022	.474
Never vs. current	---	---	---	---	---	---	.071	.0374	1.074	.998	1.155	.058
Never vs. missing	---	---	---	---	---	---	4.880E-5	.0287	1.000	.945	1.058	1.000
Diabetes	---	---	---	---	---	---	.071	.0168	1.073	1.038	1.109	<.001***
COPD	---	---	---	---	---	---	.050	.0207	1.051	1.010	1.095	.015*
CAD & CHF	---	---	---	---	---	---	.088	.0165	1.092	1.057	1.128	<.001***
Chronic Renal Failure	---	---	---	---	---	---	.072	.0173	1.075	1.039	1.112	<.001***
Cancer	---	---	---	---	---	---	.144	.0288	1.155	1.091	1.222	<.001***
Cirrhosis	---	---	---	---	---	---	.151	.0698	1.163	1.015	1.334	.030*
Lipid Metabolism Disorders	---	---	---	---	---	---	-.037	.0166	.963	.933	.995	.026*
**Alzheimer's Disease**	.189	.0313	1.209	1.137	1.285	<.001***	.193	.0335	1.213	1.135	1.295	<.001***
Sex	---	---	---	---	---	---	.090	.0331	1.094	1.025	1.167	.007**
Race[a]	---	---	---	---	---	---						
Health System[a]	---	---	---	---	---	---						
COVID-19 vaccination	---	---	---	---	---	---						
0 vs. 1	---	---	---	---	---	---	-.034	.0796	.967	.827	1.130	.673
0 vs. 2	---	---	---	---	---	---	-.112	.0481	.894	.814	.982	.020*

0 vs. 3	---	---	---	---	---	---	-.212	.0826	.809	.688	.951	.010*
Smoking	---	---	---	---	---	---						
Never vs. former	---	---	---	---	---	---	-.036	.0365	.965	.898	1.036	.328
Never vs. current	---	---	---	---	---	---	-.004	.0882	.996	.838	1.184	.965
Never vs. missing	---	---	---	---	---	---	.049	.0595	1.050	.934	1.180	.412
Diabetes	---	---	---	---	---	---	.097	.0360	1.102	1.027	1.183	.007**
COPD	---	---	---	---	---	---	.013	.0452	1.013	.927	1.107	.770
CAD & CHF	---	---	---	---	---	---	.084	.0353	1.087	1.014	1.165	.018*
Chronic Renal Failure	---	---	---	---	---	---	.073	.0371	1.076	1.000	1.157	.049*
Cancer	---	---	---	---	---	---	.134	.0624	1.143	1.012	1.292	.032*
Cirrhosis	---	---	---	---	---	---	.060	.1639	1.062	.770	1.464	.716
Lipid Metabolism Disorders	---	---	---	---	---	---	-.043	.0348	.958	.895	1.026	.219
**Vascular Dementia**	.531	.0457	1.701	1.556	1.861	.000***	.451	.0518	1.570	1.418	1.738	<.001***
Sex	---	---	---	---	---	---	.074	.0479	1.077	.980	1.183	.123
Race[a]	---	---	---	---	---	---						
Health System[a]	---	---	---	---	---	---						
COVID-19 vaccination	---	---	---	---	---	---						
0 vs. 1	---	---	---	---	---	---	-.147	.1184	.864	.685	1.089	.216
0 vs. 2	---	---	---	---	---	---	-.160	.0712	.852	.741	.979	.024*
0 vs. 3	---	---	---	---	---	---	-.480	.1383	.619	.472	.812	<.001***
Smoking	---	---	---	---	---	---						
Never vs. former	---	---	---	---	---	---	-.076	.0532	.927	.835	1.029	.155
Never vs. current	---	---	---	---	---	---	-.001	.0989	.999	.823	1.213	.993
Never vs. missing	---	---	---	---	---	---	.015	.0994	1.015	.835	1.234	.880
Diabetes	---	---	---	---	---	---	.067	.0510	1.069	.967	1.181	.192
COPD	---	---	---	---	---	---	-.026	.0615	.974	.864	1.099	.670
CAD & CHF	---	---	---	---	---	---	.150	.0512	1.162	1.051	1.285	.003**
Chronic Renal Failure	---	---	---	---	---	---	.011	.0530	1.011	.911	1.121	.839
Cancer	---	---	---	---	---	---	.244	.0919	1.276	1.066	1.527	.008**
Cirrhosis	---	---	---	---	---	---	.302	.2027	1.353	.909	2.013	.136

Lipid Metabolism Disorders	---	---	---	---	---	---	-.016	.0522	.984	.888	1.090	.754

* *p* < .05, ** *p* < .01, *** *p* < .001

Note. *B* = Unstandardized Beta; IRR = Incidence-Rate Ratio; 95% CI = 95% Confidence Interval; LL = Lower Limit; UL = Upper Limit; All Dementia: coded as 0 = no dementia, 1 = dementia; Alzheimer's disease: coded as 0 = no dementia of any kind, 1 = Alzheimer's disease; Vascular Dementia: coded as 0 = no dementia of any kind, 1 = Vascular Dementia; COPD = chronic obstructive pulmonary disease; CAD & CHF = coronary artery disease and congestive heart failure. [a]Race and Health System were each included as categorical variables in analyses, but presentation of the comparisons of subcategories was not included in tables for space purposes. These results can be obtained from the author by request.

Figure S1. Map of Health Systems.

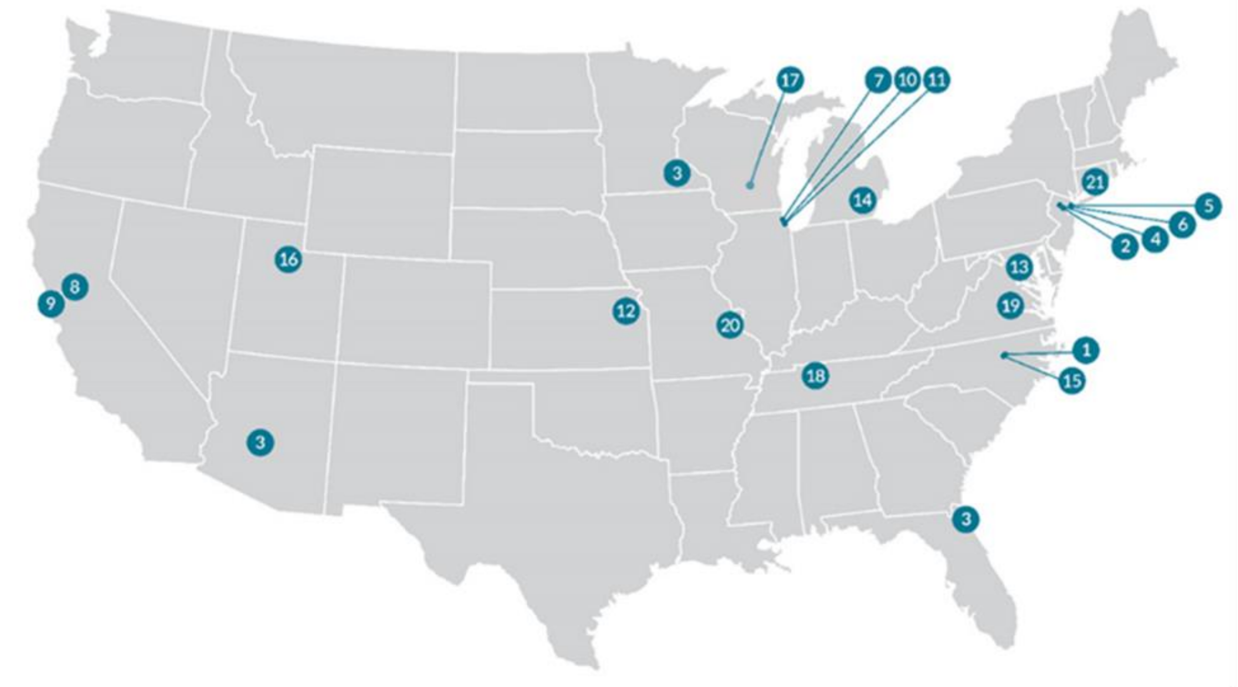

1. Duke University	2. Hackensack Meridian Health	3. Mayo Clinic (3 locations: Phoenix, AZ, Jacksonville, FL, Rochester MN)
4. Memorial Sloan Kettering Cancer Center	5. Mount Sinai	6. New York University
7. Northwestern University	8. University of California-Davis	9. University of California-San Francisco
10. University of Chicago	11. University of Illinois at Chicago	12. University of Kansas
13. University of Maryland	14. University of Michigan	15. University of North Carolina
16. University of Utah	17. University of Wisconsin	18. Vanderbilt University
19. Virginia Commonwealth University	20. Washington University	21. Yale University

Figure S2. Distribution of Dementia Subtypes by Health System.

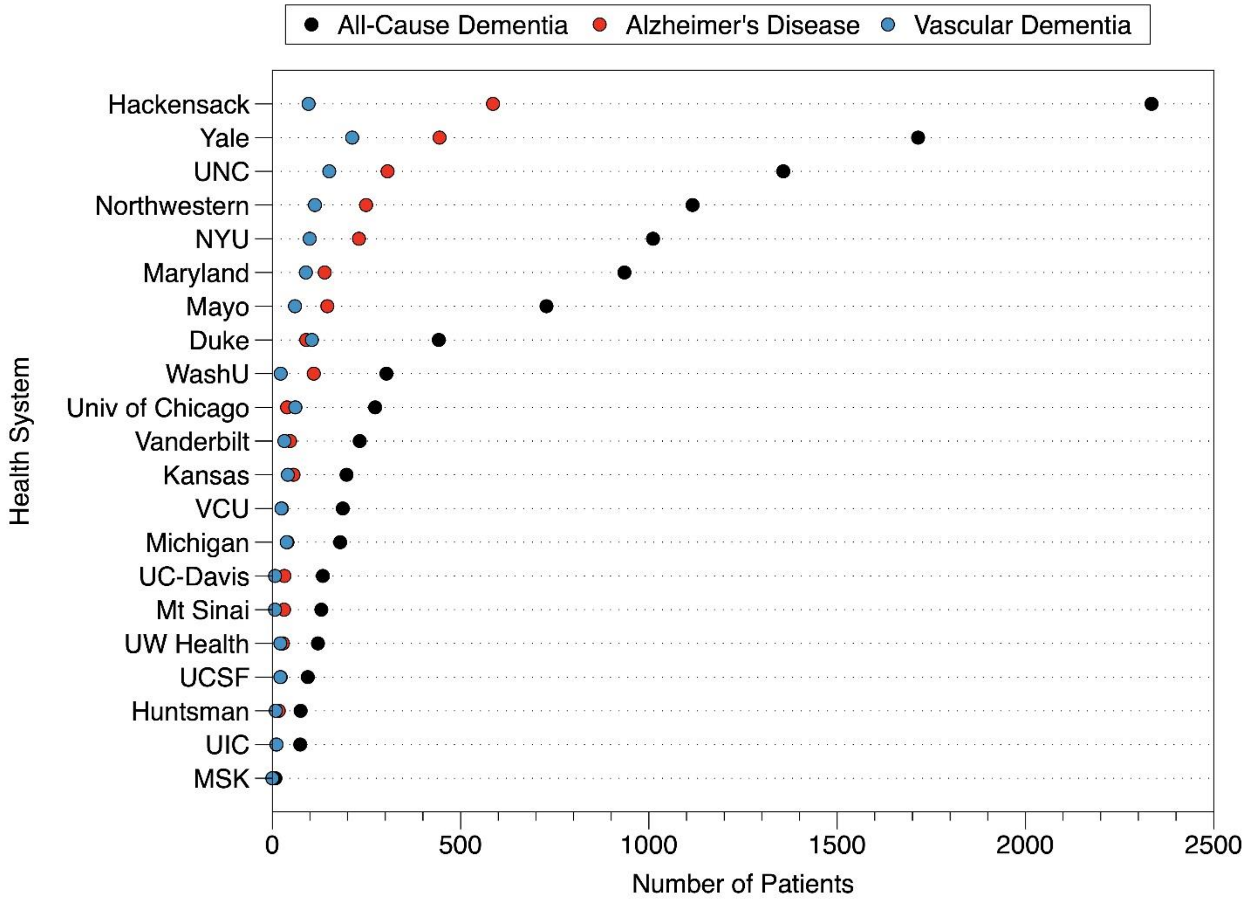

Figure S3. Race by Vascular Dementia Interaction for ICU Admission.

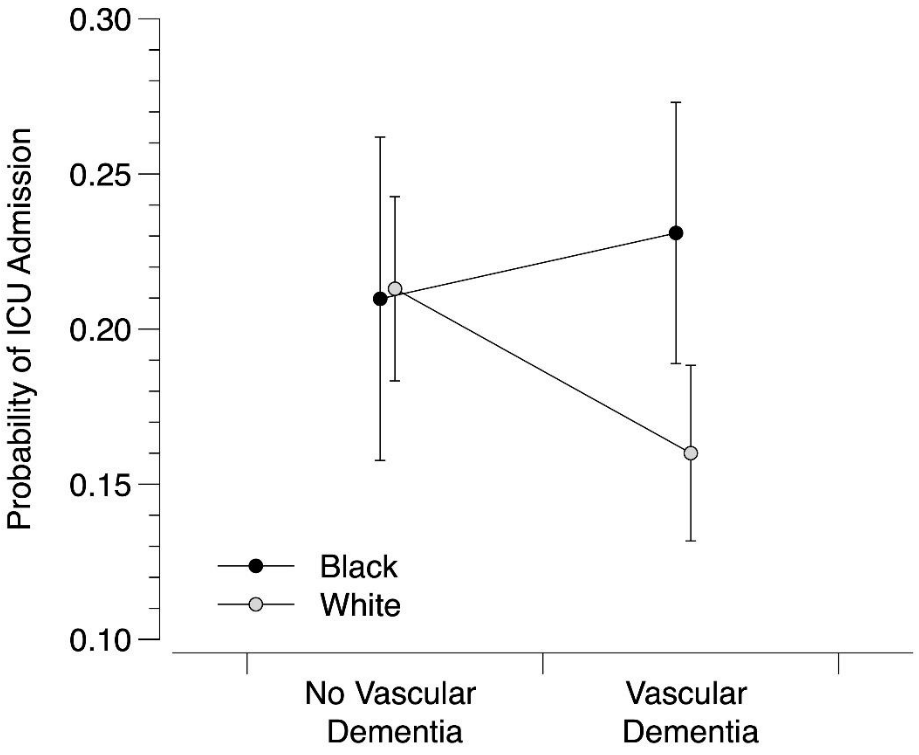

Note. Error bars represent 95 percent confidence intervals.

Figure S4. Sex by All-Cause Dementia Interaction for Hospital Stay Duration.

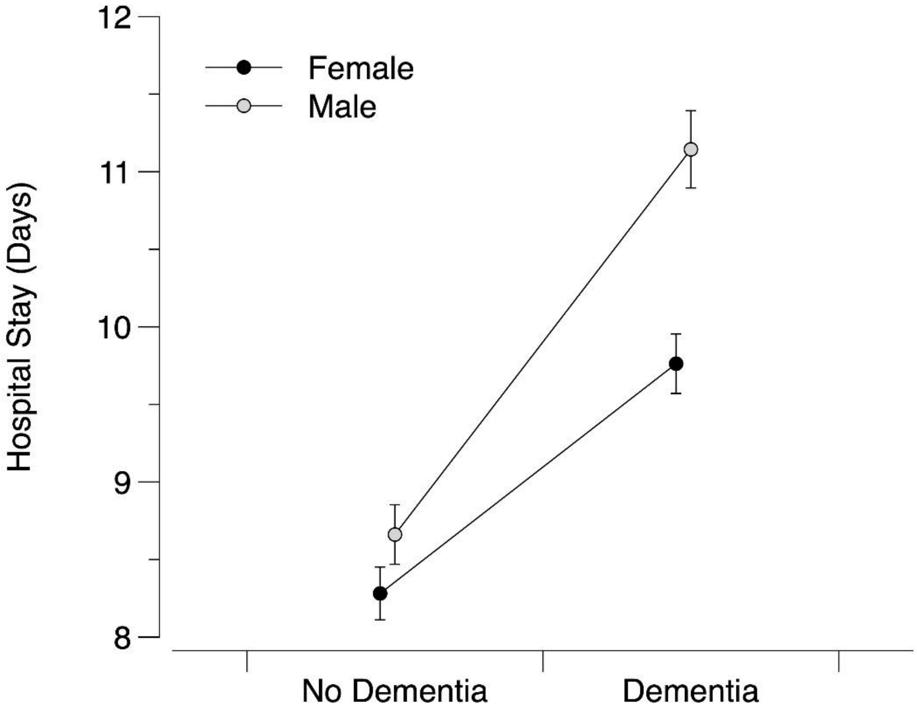

Note. Error bars represent 95 percent confidence intervals.

Figure S5. Sex by Alzheimer's Disease Interaction for Hospital Stay Duration.

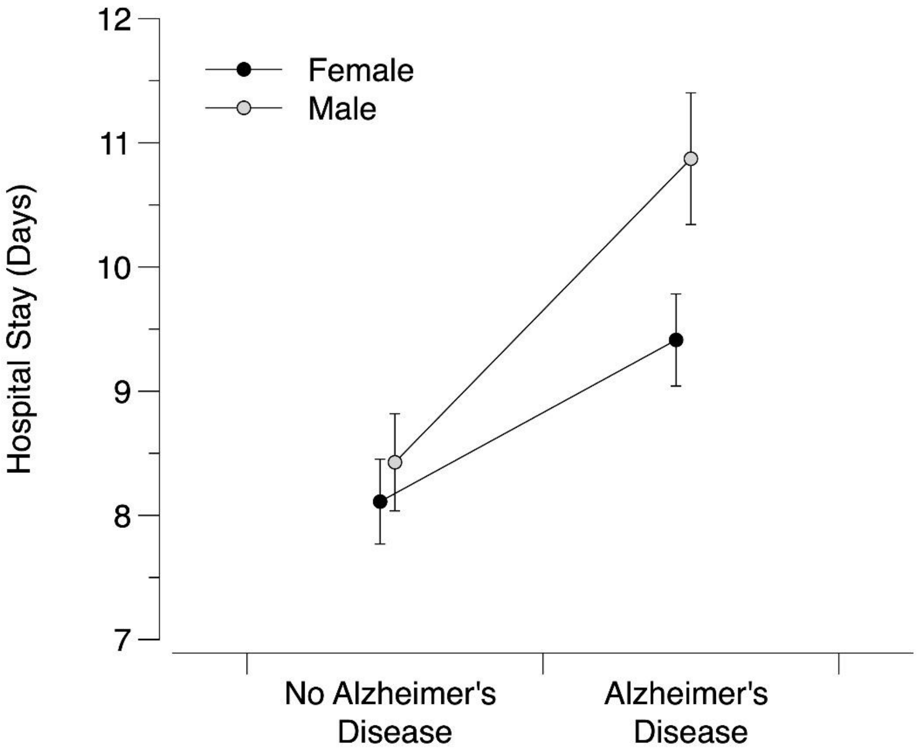

Note. Error bars represent 95 percent confidence intervals.

Figure S6. Sex by Vascular Dementia Interaction for Hospital Stay Duration.

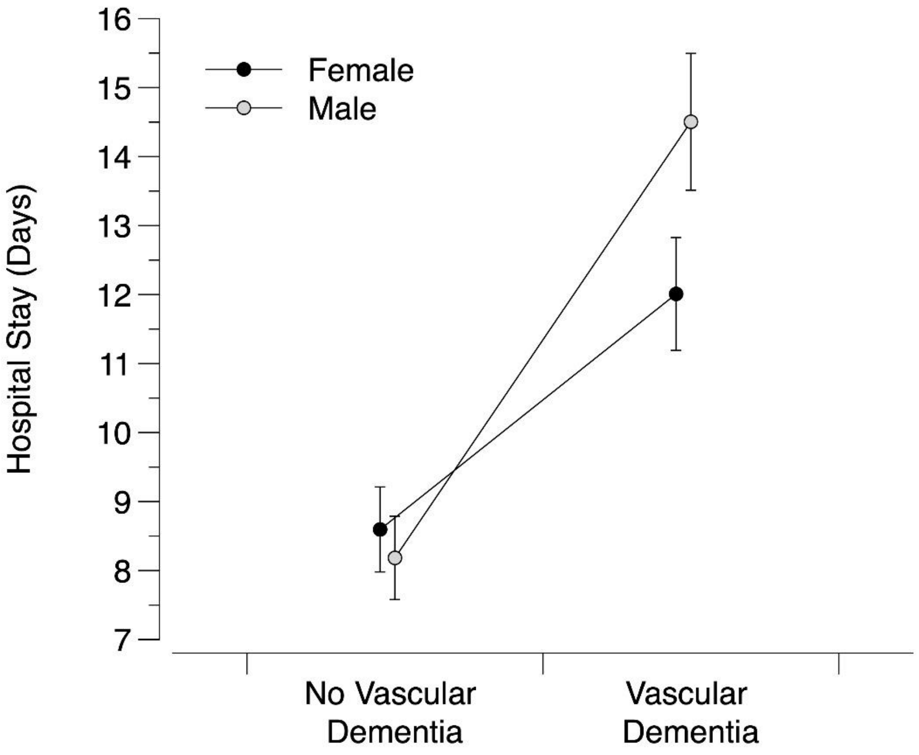

Note. Error bars represent 95 percent confidence intervals.

Figure S7. Race by Vascular Dementia Interaction for Hospital Stay Duration.

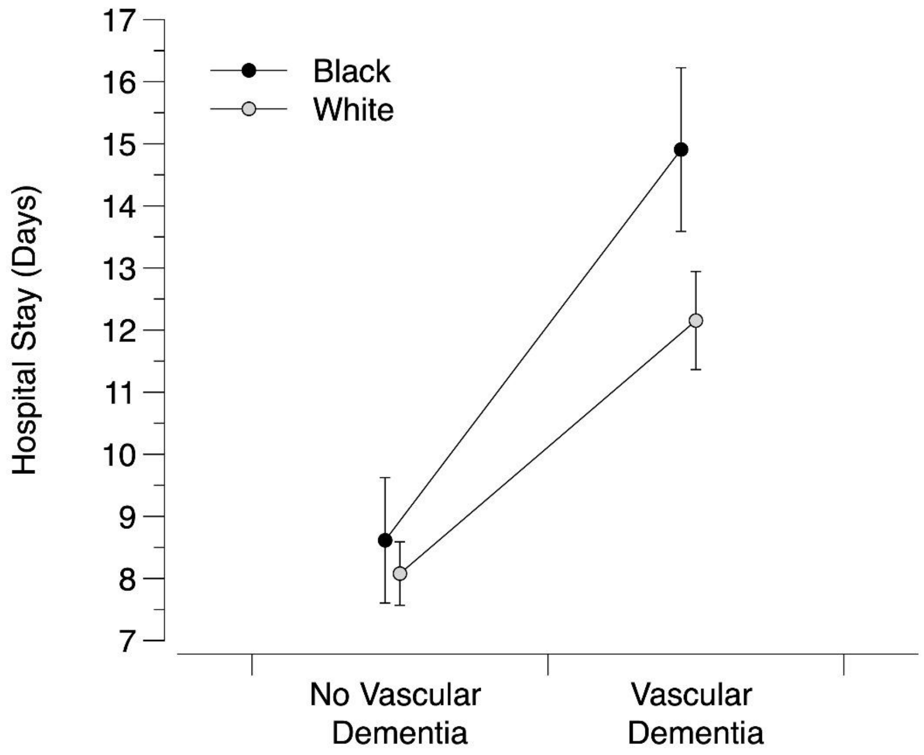

Note. Error bars represent 95 percent confidence intervals.
